# A Toolbox for Translational Research on Beta Cell Function: Commentary on: “A Dual Reporter EndoC-βH1 Human Beta Cell Line for Efficient Quantification of Calcium Flux and Insulin Secretion.”

**DOI:** 10.1210/endocr/bqaa025

**Published:** 2020-02-27

**Authors:** Torben Schulze, Ingo Rustenbeck

**Affiliations:** Institute of Pharmacology and Toxicology, University of Braunschweig, Braunschweig, Germany

**Keywords:** pancreatic beta cell, insulin secretion, translational research

The public perception of diabetes has long been dominated by type 1 diabetes, because of the often severe symptoms at the clinical onset, the lifelong dependence on insulin injections, and the mostly young age at which this disease begins. Nowadays, this form of diabetes, although still increasing, makes up less than 10% of all cases. Type 2 diabetes in contrast, once considered to be of marginal relevance, has developed into a worldwide threat to human health and health systems. The limited efficiency of current therapies requires efforts by the pharmaceutical industry to develop new and affordable drugs.

A multitude of functional deficiencies contribute to this heterogeneous disease. This fact has in principle been known for a long time, but has recently gained renewed attention by the demonstration of data-driven clustering in a large cohort of type 2 diabetes patients (1). While the relevance of insulin secretion deficiency varies between these groups, it is clear that a dysfunction of the endocrine pancreas is an independent pathogenetic factor and not only secondary to the increased workload caused by the insulin resistance. A genome-wide association study showed that most of the genes which are associated with susceptibility to type 2 diabetes concern beta cell function (2).

The diminished insulin response to glucose in type 2 diabetes is usually described to be more prominent during the first phase, but is also recognizable during the second phase. While a consensus on the basic features of insulin secretion has been reached, there is still a debate as to which signals contribute to the biphasic kinetics of insulin secretion. It is clear that depolarization-induced calcium ion (Ca^2+^) influx is required for insulin granule fusion, similar to the exocytosis of neurotransmitters. However, insulin secretion is a much slower process than neuronal signal transmission. So, the kinetics of secretion may be shaped by the velocity of glucose metabolism and by the generation of metabolites that affect the availability of granules for exocytosis, a process currently named metabolic amplification.

Research on the endocrine pancreas and specifically, on the stimulus-secretion coupling, is hampered by its organization as small cell clusters distributed throughout the exocrine pancreas. Collagenase digestion is in principle able to isolate fully functional pancreatic islets from the surrounding exocrine tissue, but this method requires considerable skill, is time-consuming and yields small amounts of tissue. Even under carefully controlled conditions, isolated islets display considerable heterogeneity, often necessitating numerous repetitions of the experiments to demonstrate significant effects. This is particularly true for freshly isolated islets but also for cultured islets.

Therefore, immortalized beta cell lines have come into use as a more readily available cell source. Currently, the most popular of these are MIN6- and INS1E-cells, both derived from rodent species. One lesson to be learnt from cell lines was that quick growth (and thus easy transfection) is inversely proportional to the maintenance of the typical features of the beta cell. Since there is evidence that the cell distribution within islets from rodents differs from that of human islets, and since the kinetics of insulin release of human and murine islets display differences (3), the generation of an immortalized beta cell line of human origin (EndoC-βH1) was a step forward (4).

In the article by Cardenas-Diaz and colleagues (5) the properties of a human beta cell line are described which was designed to permit rapid functional testing. To this end, the endoC-βH1 cell line was lentivirally transduced with a GCaMP6 Ca^2+^ sensor protein (6) and a preproinsulin-luciferase fusion protein contained in one vector. To demonstrate the ability of the transduced cells to report secretory responses they were challenged with a stimulatory glucose concentration and with a strongly depolarizing concentration of potassium chloride (KCl).

Measurement of the cytosolic Ca^2+^ concentration cannot substitute for the measurement of insulin secretion since the extent of secretion is modulated by metabolic amplification. For this reason it is an advantageous property that the triggering Ca^2+^ signal can be monitored by fluorescence and at the same time the secretion by the luminescence of the released luciferase. As compared to the earlier days of islet research, the commercial availability of insulin or C-peptide enzyme-linked immunosorbent assays (ELISAs) has made the measurement of secretion much more flexible. But ELISA is not an ideal technique for high throughput measurements and, when a high time resolution is required, the use of commercial ELISAs becomes forbiddingly expensive.

Thus, the demonstration that the luciferase luminescence correlates with the released C-peptide levels opens up the possibility to screen entire libraries of compounds for their effects on beta cell function. Another possibility, not discussed by the authors, would be to continuously monitor secretion by the constant addition of luciferin and adenosine triphosphate (ATP), analogous to the postcolumn derivatization in chromatography. The precise mechanisms of insulin exocytosis, however, is likely beyond the scope of applications, given the more than 10-fold higher molecular mass of the luciferase molecule (62 kD). Nevertheless, the dual-reporter cell line may also be of value for basic research. The loss of glucose- but not KCl-stimulated Ca^2+^ increase and insulin secretion as a consequence of CRISPR-Cas9-mediated knockout of *PDX1* serves as an example that the relevance of candidate genes in human beta cells can be tested by use of this system.

The limitations are those which generally apply to insulin-secreting cell lines. The biosynthetic activity required for cell division and growth may come at the expense of metabolic signal recognition. Stability of the characteristics from one passage to the next may be an issue. Furthermore, the islet consists of multiple cell types which act in concert. So, for basic research there is still a case for the use of gently isolated or reconstituted islets, in particular when used in conjunction with microfluidic perifusion systems (7).

In conclusion, the system presented by Cardenas-Diaz and colleagues is particularly well-suited to check the effects of pharmacological agents or of genetic manipulations on a large scale with limited cost, which should be of considerable interest for the pharmaceutical industry or translational research in general.

## Abbreviations

Ca^2+^: calcium ion
ELISA: enzyme-linked immunosorbent assay

## Additional Information

***Disclosure Summary:*** The authors declare no conflict of interest.

***Data Availability.*** Data sharing is not applicable to this article as no datasets were generated or analyzed during the current study.
